# Th2-Immune Polarizing and Anti-Inflammatory Properties of Insulin Are Not Effective in Type 2 Diabetic Pregnancy

**DOI:** 10.1155/2020/2038746

**Published:** 2020-06-15

**Authors:** Adnette Fagninou, Magloire Pandoua Nekoua, Darius Sossou, Kabirou Moutairou, Nadine Fievet, Akadiri Yessoufou

**Affiliations:** ^1^Faculty of Sciences and Technology (FAST), University of Abomey-Calavi, Institute of Applied Biomedical Sciences (ISBA), Laboratory of Cell Biology and Physiology, 01 BP 526 Cotonou, Benin; ^2^Center for Study and Research on Malaria Associated with Pregnancy and Childhood (CERPAGE) and IRD-UMR261, Cotonou, Benin

## Abstract

**Background:**

The implication of the immune system in the physiopathology of pregnancy complicated by diabetes has been reported. Here, we investigated the effects of insulin treatment on the frequencies of immune cell subpopulations as well as T cell-derived cytokines in type 2 diabetic (T2D) pregnancy compared to gestational diabetes mellitus (GDM).

**Methods:**

Fifteen (15) women with GDM, twenty (20) insulin-treated T2D pregnant women, and twenty-five (25) pregnant controls were selected. Immune cell subpopulation frequencies were determined in blood using flow cytometry. The proliferative capacity of T cells was performed, and serum and cell culture supernatant cytokine levels were also quantified.

**Results:**

The frequencies of total CD3+ and CD4+ T cells and nonclassical monocytes significantly increased in insulin-treated T2D pregnant women compared to pregnant controls. The proportions of CD4+ T cells as well as B cells were significantly higher in women with GDM than in pregnant controls. GDM was associated with high frequencies of total CD3+ and CD4+ T cells and B cell expansion, suggesting a concomitant activation of cellular and humoral immunity. Concomitantly, Th1/Th2 ratio, determined as IFN-*γ*/IL-4, was shifted towards Th1 phenotype in women with GDM and insulin-treated T2D pregnant women. Besides, isolated T cells elicited similar proliferative capacity in the three groups of women. Insulin-treated T2D pregnant women and women with GDM exhibited a low serum IL-10 level, without any change in the number of Treg cells.

**Conclusion:**

Our study showed that, despite insulin treatment, pregnant women with T2D displayed a proinflammatory status consistent with high proportions of CD3+ and CD4+ T cells, upregulation of Th1 cytokines, and low IL-10 production, suggesting a reduced immune-suppressive activity of regulatory T cells. However, GDM, although associated with proinflammatory status, has shown increased humoral immunity consistent with high proportion of CD19+ B cells. Thus, the lack of response to insulin in diabetes during pregnancy and clinical implications of these immunological parameters deserves further investigations.

## 1. Introduction

Several studies have gained interest into the immunopathology and physiopathology of gestational diabetes mellitus (GDM), type 1 or type 2 diabetic pregnancy [1–4]. However, few is known about the implication of immune cells in pregnancy complicated by diabetes. In a recent study, we have investigated the modulation of immunological parameters in insulin-treated type 2 diabetic patients, without pregnancy [5]. However, not much is known how insulin treatment can modulate immune status in GDM and T2D during pregnancy. Gestational diabetes mellitus (GDM) is defined as glucose intolerance and increased insulin resistance which occurs most often during the second or third trimester of pregnancy while type 2 diabetes (T2D) is characterized by defects in insulin secretion and by impaired insulin sensitivity [6, 7]. The physiopathology of GDM and T2D appears to be similar, so that GDM may reflect an early stage of T2D occurring in the context of pregnancy [8]. GDM and T2D are known as a state of chronic systemic inflammation mediated by proinflammatory cytokines which are involved in the development of insulin resistance and in the increase of miscarriage rate during pregnancy [1, 9–12]. Thus, patients with GDM have an increased risk of developing T2D after their pregnancy [13, 14]. Moreover, T2D is currently regarded as a chronic inflammatory disease associated with proinflammatory cytokine production and immune cell activities, including B and T cell subsets as pathogenic mediators [15–17]. But so far, not much literature exists about circulating immune cell distribution in pregnant women with T2D and GDM. Nonetheless, some studies showed that GDM is not only due to metabolic disturbances during pregnancy but also by a state of low-grade systemic inflammation [18]. This dysregulation of the immune system is characterized by an altered profile of monocytes and regulatory T cells in peripheral blood and an imbalance between T helper 1 (Th1) and 2 (Th2) cells favoring proinflammatory responses [12, 19–21]. As the hyperglycemia and disrupted immunological adaptations during pregnancy are responsible for increased maternal-fetal morbidity, as well as the short- and long-term complications in mothers and offspring [1, 22, 23], improving glycemic control by insulin therapy may substantially reduce the risks of these maternal and neonatal outcomes of diabetes in pregnancy [24–26]. In fact, insulin not only prevents the deleterious effects of hyperglycemia by improving the anabolism of glucose, proteins, and lipids but also promotes a systemic anti-inflammatory response through the reduction of proinflammatory cytokines. Conversely, the increase of anti-inflammatory cytokines supports a successful pregnancy due to their immunosuppressive effects [27–30]. Moreover, many studies have shown that insulin can modulate *in vitro* the proliferation, differentiation, metabolism, and immune functions of neutrophils, monocytes, macrophages, effector, and regulatory T cells [31–33]. In a recent previous study, we have showed, in type 2 diabetes without pregnancy, that insulin treatment can modulate immunological parameters, through immune cell subpopulation and cytokines, and confer to these patients a protective Th2 phenotype [5]. However, despite some progress in understanding the immunophysiopathology of GDM and T2D during pregnancy, there still exists some controversies about the profile of immune parameters in diabetes during pregnancy. Moreover, it remains unclear whether insulin treatment can modulate the immune status of pregnant women with GDM and T2D through the pattern of immune cell subtypes and cytokines. Therefore, the present study was undertaken to investigate the effect of insulin treatment on the frequencies of leucocyte subpopulations along with the profile of T cell-derived cytokines in pregnant women with T2D in comparison with women with GDM and healthy pregnant women.

## 2. Material and Methods

### 2.1. Subjects and Diabetes Diagnosis in Pregnant Women

For first general selection of participants in this cross-sectional study, a total of one hundred and seventy-five (175) pregnant women were enrolled by specialist clinicians of the Department of Obstetrics and Gynecology of three national hospital centers in southern Benin. Based on the exclusion criteria (please see below), one hundred and fifty-three (153) pregnant women, aged from 19 to 43 years, were selected and then screened for GDM (please see below the detailed protocol). Consequently, fifteen (15) pregnant women were found as positive for GDM which represent 9.80% of total. Among women negative for GDM, twenty-five (25) age-matched and body mass index-matched pregnant women were selected and considered as the control group. Pregnant women with preexisting insulin-treated T2D were separately selected in the population of women already monitored by the clinicians at the department of obstetrics and gynecology of these hospital centers. The number of twenty (20) insulin-treated T2D pregnant women corresponds to the mean of the number of women with GDM (15) and the number of control pregnant (25) women. The size of each group, fifteen (15) women with GDM, twenty (20) pregnant women with insulin-treated T2D, and twenty-five (25) pregnant controls, was appropriate for statistics. All selected participants were then submitted to blood collection for biochemical and immunological assays.

GDM was diagnosed in pregnant women by an oral glucose tolerance test (OGTT), according to the criteria of the International Association of Diabetes and Pregnancy Study Group (IADPSG). Briefly, women between 24 and 28 weeks of gestation, after overnight fasting, were given 75 g of glucose. Subjects were declared as positive for GDM when overnight fasting plasma glucose was ≥92 mg/dL (5.1 mmol/L), or 1 h OGTT plasma glucose level was ≥180 mg/dL (10.0 mmol/L), or 2 h OGTT plasma glucose level was ≥153 mg/dL (8.5 mmol/L) [34–36]. Pregnant women with T2D were long-established diabetic patients (disease duration = 3.4 ± 2.1 years) diagnosed according to the criteria of the American Diabetes Association [37] and were on insulin treatment.

Exclusion criteria included clinical coronary artery disease, renal and hepatic diseases, and clinical signs of infectious disease, hepatitis B, hepatitis C, HIV, and malaria infection after blood sample tests. Subjects were included after informed and written consent. The study was conducted in accordance with the Declaration of Helsinki (1964) (as revised in Edinburgh 2000) and was approved by the Ethics Committee on Research of the Institute of Applied Biomedical Sciences of Cotonou, Benin, under the number Dec.n°100/CER/ISBA-2016.

### 2.2. Blood Samples

In women with GDM, blood samples were collected immediately after diagnostic of GDM, between 24 and 28 weeks of gestation, and before any treatment. In pregnant control women as well as in insulin-treated pregnant women with T2D, blood samples were collected between 24 and 28 weeks of gestation. A fasting whole blood sample was collected by venipuncture from each woman into sterile vacuum blood collection tubes (Vacutainer System, Becton Dickinson, CA, USA) containing either ethylenediaminetetraacetic acid (EDTA) or fluoride oxalate or nothing. EDTA tube was used for immune cell subtypes phenotyping within 2 h after sampling. Glycosylated hemoglobin (HbA1c) levels were determined in the whole blood. HbA1c concentration was calculated using a percentage of total hemoglobin, according to the manufacturer's instructions (Ref. 41190, Labkit Chemelex SA, Barcelona, Spain). Plasma from blood collected in fluoride oxalate tubes was immediately used for glucose determinations by a glucose oxidase method using a glucose analyzer (Beckman Instruments). Serum was obtained by low-speed centrifugation (1000 g/20 min), distributed in aliquots and frozen at −80°C for the measurement of cytokine concentrations.

### 2.3. Immune Cell Phenotyping

**Figure 1:**
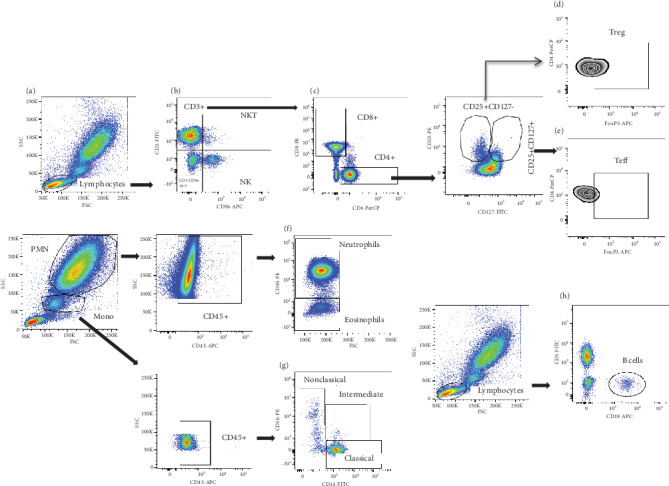
Cytometry-based gating strategies. Total lymphocytes (a) were gated to identify NK cells (CD3-CD56+) and NKT cells (CD3+CD56+) (b). CD4+ and CD8+ cells were gated from CD3 T cells (c). Regulatory T cells (d) and effector T cells (e) were, respectively, gated from CD4+CD25+CD127- and CD4+CD25+CD127+ cells. Neutrophil and eosinophil cells were identified based on CD16 from CD45+ cells in polymorphonuclear (PMN) cells (f). The nonclassical (CD16++CD14+), intermediate (CD14++CD16+), and classical (CD14++CD16-) monocytes were gated from CD45+ in total monocyte region (g). Total B cells were gated from lymphocytes (h).

The following monoclonal antibody combinations (mAbs) purchased from BD Pharmingen (France) were used to evaluate the frequencies of innate and adaptive immunity cells in whole blood: anti-CD3-FITC/anti-CD4-PerCP/anti-CD8-PE/anti-CD25-PE/anti-CD127-FITC/anti-FoxP3-APC for T cell staining, anti-CD19-FITC for B lymphocytes, anti-CD3-FITC/anti-CD56-APC for NK and NKT cells, anti CD45-APC/anti-CD14-FITC/CD16-PE for monocytes, and anti-CD45-APC/anti-CD16-PE for polynuclear cells, according to the manufacturer's instructions. Briefly, whole blood was stained with appropriate combination of specific mAbs for 45 min at 4°C in the dark. After erythrocyte lysing with FACS lysing solution (BD Pharmingen), cells were washed twice with FACS buffer. Regulatory T (Treg) cells were labelled by double staining. Anti-CD4-PerCP, anti-CD25-PE, and anti-CD127-FITC were used for Treg cell membrane labelling. Anti-FoxP3-APC was used for intracellular staining of FoxP3 in Treg cells after permeabilization and fixation with BD Cytoperm/Cytofix for 20 min at 4°C. Cells were then washed with Cytoperm/Wash and resuspended in 200 *μ*L of PBS 1x. The stained cells were acquired using a FACSCanto II flow cytometer (BD Pharmingen, France) and analyzed using FlowJo version V 10.6.1. gating strategies shown in Figure 1.

### 2.4. PBMC Isolation and T Cell Proliferation Assay

Peripheral blood mononuclear cells (PBMCs) were isolated from peripheral blood collected in tubes containing EDTA by Ficoll-Paque™PLUS (GE Healthcare, Vélizy-Villacoublay, France) gradient centrifugation (30 min at 500×g, 20°C). Cells were then suspended in RPMI-1640 culture medium (Gibco BRL, Thermo Fischer Scientific, Villebon sur Yvette, France) supplemented with 10% of complement-inactivated fetal calf serum (Invitrogen, France), 1% of nonessential amino acids (Invitrogen, France), 50 *μ*g/mL of penicillin and streptomycin (Invitrogen, France), and 2 mM of L-glutamine (Invitrogen, France). The concentration of viable cells was assessed by trypan blue staining. Cells were distributed in quadruplicate at a concentration of 10^5^ per well in a Falcon polystyrene 96-well plate (Thermo Fisher Scientific, Illkirch-Graffenstaden, France) and were cultured either in the absence or in the presence of 5 *μ*g/mL of PHA (Sigma Chemical Company, MO, USA) or of anti-CD3/anti-CD28 (BD Pharmingen, France) at a respective dose of 1 *μ*g/mL and 2 *μ*g/mL. After 6 days of culture at 37°C in a humid environment with 5% CO_2_, the supernatants were collected and stored at -80°C for cytokine measurement (IFN-*γ*, IL-2, IL-4, and IL-10) and T cell proliferation was evaluated through cell count using the trypan blue exclusion test on microscopy [38].

### 2.5. In Vivo and In Vitro Cytokine Level Determinations

Cytokines were quantified in serum samples and in PBMC culture supernatants using enzyme-linked immunosorbent assay kits (BioLegend human Th1/Th2 ELISA MAX™ Deluxe, San Diego, CA, USA), according to the manufacturer's instructions. The minimum detectable concentrations were 4 pg/mL (standard ranges = 7.8–500 pg/mL) for IL-2 and IFN-*γ* and 2 pg/mL (standard ranges = 3.9–250 pg/mL) for IL-4 and IL-10.

### 2.6. Statistical Analysis

Data analyses were performed using GraphPad Prism 6.0 (GraphPad Inc., CA, USA). Values are means ± SD or medians ± IQR. The Kruskal-Wallis test, followed by Dunn's multiple comparison test, was used to analyze differences between the three groups (women with GDM, pregnant women with T2D, and healthy pregnant women). The Mann–Whitney *U* test was also used when appropriate. *p* values < 0.05 were considered to indicate statistically significant differences.

## 3. Results

### 3.1. Anthropometric and Biochemical Data of Subjects

Anthropometric and biochemical data of the participants appear in Table 1. There was no significant difference in age and gestational age between the three groups of participants. HbA1c values did not differ between the three groups of pregnant women. As compared to control pregnant women, insulin-treated T2D pregnant women showed a normal level of fasting glycemia. However, fasting glycemia was significantly higher in women with GDM than in either insulin-treated T2D pregnant women (1.23 ± 0.05 g/L *vs.*0.89 ± 0.02 g/L, *p* = 0.001) or pregnant controls (1.23 ± 0.05 g/L *vs.*0.80 ± 0.03 g/L, *p* = 0.001) (Table 1). The duration of disease was 3.4 ± 2.1 years in insulin-treated T2D pregnant women (Table 1).

### 3.2. Immune Cell Frequencies in GDM and Insulin-Treated T2D

**Figure 2:**
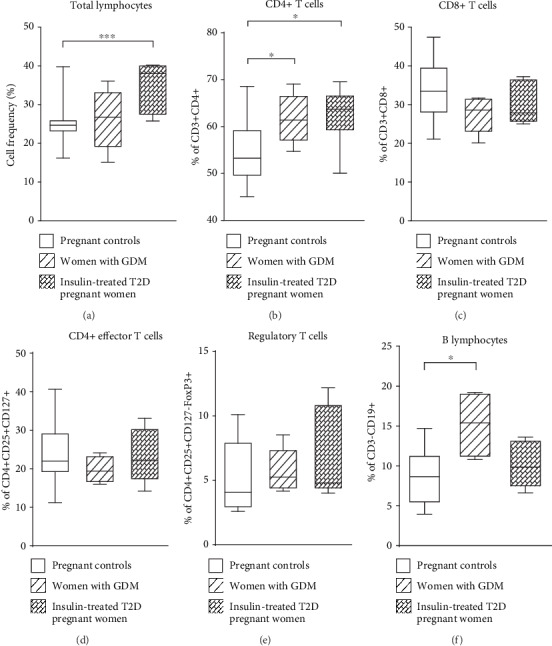
Frequencies of peripheral blood adaptative immunity cells in women with gestational diabetes mellitus (GDM), insulin-treated T2D pregnant women, and pregnant controls. Data shown as box plots representing medians (with 25th and 75th percentiles) and whiskers (10th and 90th percentiles) of immune cell subset frequencies: (a) total lymphocytes, (b) CD4+ T cells, (c) CD8+ T cells, (d) effector T cells (CD4+CD25+CD127+), (e) regulatory T cells (CD4+CD25+CD127-FoxP3+), (f) CD19+ B lymphocytes from *n* = 15 women with GDM, *n* = 20 insulin-treated T2D pregnant women, and *n* = 25 pregnant controls. The statistical differences were determined using the nonparametric Kruskal-Wallis test. ^∗^*p* < 0.05; ^∗∗^*p* < 0.01; ^∗∗∗^*p* < 0.001.

**Figure 3:**
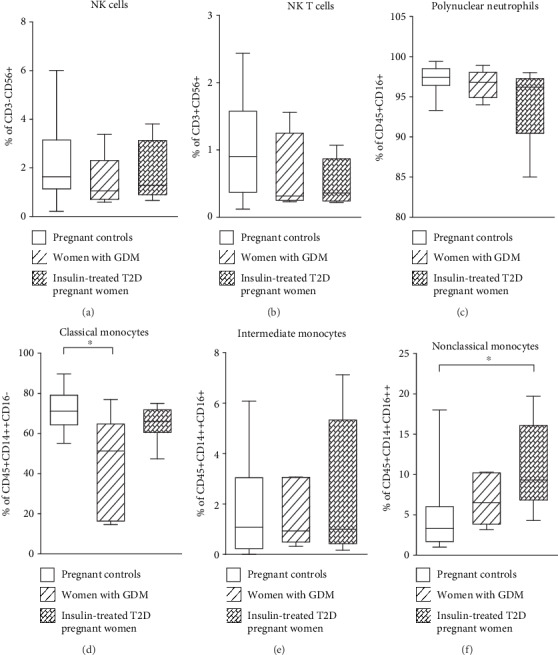
Frequencies of peripheral blood innate immunity cells in women with gestationnel diabetes mellitus (GDM), insulin-treated T2D pregnant women and pregnant controls. Data shown as box plots representing medians (with 25th and 75th percentiles) and whiskers (with 10th and 90th percentiles) of immune cell subset frequencies: (a) NK cells (CD3-CD56+), (b) NKT cells (CD3+CD56+), (c) polynuclear neutrophils (CD45+CD16+), (d) classical monocytes (CD45+CD14++CD16-), (e) intermediate monocytes (CD45+CD14++CD16+), (f) nonclassical monocytes (CD45+CD14+CD16++) from *n* = 15 women with GDM, *n* = 20 insulin-treated T2D pregnant women, and *n* = 25 pregnant controls. The statistical differences were determined using the nonparametric Kruskal-Wallis test. ^∗^*p* < 0.05; ^∗∗^*p* < 0.01; ^∗∗∗^*p* < 0.001.

The frequencies of total CD3+ and CD4+ T lymphocytes were significantly higher in insulin-treated pregnant women with T2D compared to pregnant controls (*p* = 0.0008 and *p* = 0.019, respectively) (Figures 2(a) and 2(b)). The proportions of CD4+ T cells as well as B cells were significantly higher in women with GDM than in pregnant controls (*p* = 0.043 and *p* = 0.017, respectively) (Figures 2(b) and 2(f)). The frequencies of CD8+ T cells, effector T cells, and regulatory T cells did not significantly change between the three groups (Figures 2(c)–2(e)). Besides, the frequencies of NK cells, NKT cells, intermediate monocytes, and polynuclear neutrophils did not differ between the three groups (Figures 3(a)–3(c) and 3(e)). In contrast, the frequency of classical monocytes was significantly lower in women with GDM than in pregnant controls (*p* = 0.022) (Figure 3(d)) and nonclassical monocytes were increased in insulin-treated pregnant women with T2D compared to those in pregnant controls (*p* = 0.011) (Figure 3(f)).

### 3.3. Proliferative Capacity of T Cells in GDM and Insulin-Treated T2D

**Figure 4:**
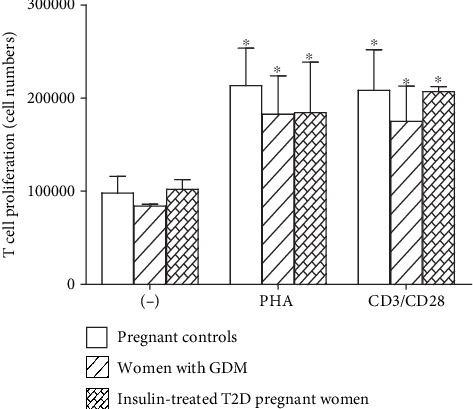
Lymphocyte proliferation of women with gestational diabetes mellitus (GDM), insulin-treated T2D pregnant women, and pregnant controls. PBMCs were cultured 6 days either in the absence or in the presence of 5 *μ*g/mL of PHA or combination of 1 *μ*g/mL of anti-CD3 and 2 *μ*g/mL of anti-CD28. Values are means ± SEM of four determinations.

Under anti-CD3/anti-CD28 and PHA stimulation, the proliferative abilities of T cells were similar between the three groups, women with GDM (*p* = 0.035 and *p* = 0.04), insulin-treated T2D pregnant women (*p* = 0.028 and *p* = 0.012), and pregnant control women (*p* = 0.001 and *p* = 0.001), as compared to unstimulated cells (Figure 4). However, it is interesting to note that the mitogens (antiCD3/CD28 and PHA) have shown the same power of stimulation on T cells from three groups of pregnant women (Figure 4).

### 3.4. Cytokine Profiles in Serum and in T Cell Culture Supernatants

**Figure 5:**
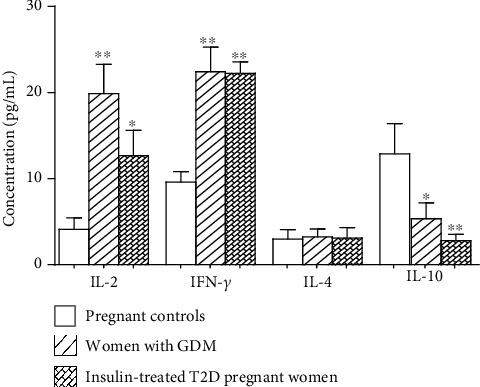
Serum concentrations of cytokines Th1 (IL-2, INF-*γ*), Th2 (IL-4), and IL-10 in women with gestational diabetes mellitus (GDM), insulin-treated T2D pregnant women, and pregnant controls. Values are means ± SD. *n* = 15 women with GDM, *n* = 20 insulin-treated T2D pregnant women, and *n* = 25 pregnant controls. ^∗^*p* values indicate significant difference between pregnant controls and pregnant diabetic patients (GDM and T2D).

As compared to control pregnant women, serum IL-2 and IFN-*γ* increased (*p* = 0.002 and *p* = 0.029) while IL-10 diminished (*p* = 0.017 and *p* = 0.002) in women with GDM and insulin-treated T2D pregnant woman (Figure 5). However, IL-4 concentrations did not differ between the three groups of pregnant women. Th1/Th2 ratios determined as IL-2/IL-4 and IFN-*γ*/IL-4 were shifted towards Th1 phenotype in women with GDM and insulin-treated T2D pregnant women (Table 2).

**Figure 6:**
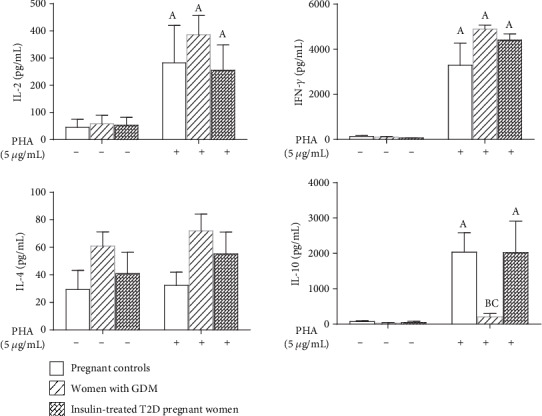
Cytokine level in the supernatants of PBMC culture under PHA stimulation. *n* = 15 women with gestational diabetes mellitus (GDM), *n* = 20 insulin-treated T2D pregnant women, and *n* = 25 pregnant controls. (A) (*p* < 0.05) indicates significant difference between cytokine levels in culture supernatant of PHA-stimulated cells and unstimulated cells of each group, (B) (*p* < 0.05) indicates significant difference between cytokine concentrations in culture supernatant after PHA stimulation between the pregnant controls and pregnant diabetic patients groups (GDM and DT2), and (C) (*p* < 0.05) indicates significant difference between cytokine concentrations in culture supernatant after PHA stimulation between women with GDM and pregnant controls and insulin-treated T2D pregnant women. Values are means ± SEM.

Besides, in PBMC culture supernatants, IL-2 and IFN-*γ* concentrations were significantly increased after PHA stimulation in the three groups compared to unstimulated cells (Figure 6). The IL-4 secretion level in culture supernatant did not differ in all pregnant women before and after stimulation with PHA (Figure 6). In contrast, IL-10 production in the supernatant after PHA-stimulation significantly increased in pregnant controls (*p* = 0.002) and in insulin-treated T2D pregnant women (*p* = 0.027) but not in women with GDM (Figure 6). It is worthy to note that, IL-2, IFN-*γ*, and IL-4 increased at a similar level after PHA stimulation (Figure 6).

## 4. Discussion

More and more evidence is accumulating and showing that the immune system, through immune cells and their products (cytokines and antibodies), plays a crucial role in modulating the severity of preexisting diabetes or diabetes occurring during pregnancy called gestational diabetes mellitus [39, 40]. In this context, any therapeutic intervention that could minimize the severity of diabetes during pregnancy could have an impact on the immune status of women with GDM and T2D pregnancy. Here, we investigated the effect of insulin treatment on the profile of immune cell subpopulations and their cytokines in pregnant women with T2D in comparison to women with GDM and healthy pregnant women. As far as the model of diabetes is concerned, several points are worth noting. The GDM offers the opportunity to study the early pathogenesis of T2D due to their similar pathophysiology and their interrelationship [7]. Thus, women with newly diagnosed GDM represent an ideal population model to study the immune status of pregnant women with T2D before insulin treatment. By the same way, prepregnancy care is also needed to prepare women with preexisting diabetes for pregnancy, such as improving glycemic control by insulin therapy [23].

As far as metabolic aspect is concerned, all the three groups of pregnant women, in the present study, exhibited normal levels of HbA1c, suggesting adequate glycemic control, although women with GDM elicited high fasting glycemia as compared to other groups. It has been proven that insulin therapy may improve glycemic control and reduce the risk of long-term complications in persons with diabetes [41, 42]. The high glycemia was observed in women with GDM although their normal level of glycosylated hemoglobin might be explained by the fact that these pregnant women are newly diagnosed for GDM and they have not yet been submitted to any treatment [41, 42]. However, pregnant women with type 2 diabetes who showed normal glycemia and HbA1c were still on insulin treatment during almost 3.4 ± 2.1 years of duration of disease [43, 44].

It has been proven that insulin therapy through the regulation of metabolic mechanisms and immune responses may improve glycemic control and reduce the risk of long-term complications in persons with diabetes [45, 46]. However, it remains unclear whether the control of glycemia may modulate the immune status in persons with diabetic pregnancy on insulin treatment. Very recently, we investigated the effect of glycemic control on a wide array of immune parameters in insulin-treated persons with type 1 diabetes [47]. We found that the insulin-treated persons with type 1 diabetes displayed a Th2-biased immune phenotype with a high proportion of effector CD4+ T cells and CD19+ B cells, and a downregulation of Th1 serum cytokines, irrespective of their capacity for glycemic control [47]. Moreover, we have recently conducted another study on nonpregnant person with type 2 diabetes on insulin treatment. We have observed in these patients, compared to control subjects, a shift of Th1/Th2 balance towards an anti-inflammatory Th2 phenotype [33]. This phenotype was consistent with a decrease of the percentages of total lymphocytes (CD3+) and CD8+ T-cells without any change in CD4+ T cells. However, the frequencies of effector CD4+ T cells, regulatory T cells, and B cells increased, suggesting a decrease of proinflammatory cellular immunity in nonpregnant type 2 diabetic patients treated with insulin, as compared to control subjects [33]. The present study evaluates the effects of insulin in combined context of type 2 diabetes and pregnancy, and we observed an inverse situation: increased frequencies of total CD3+ lymphocytes and CD4+ T cells, without any change in the frequencies of effector CD4+ T cells, and regulatory T (Treg) cells and B cells in insulin-treated pregnant women with T2D. Based on these observations, we may state that pregnancy did not permit insulin treatment to shift the Th1/Th2 balance to an anti-inflammatory phenotype in pregnant women with T2D [26–29, 31]. The same observations had been made in women with GDM in whom the profiles of CD3+, CD4+, and CD8+ T and Treg cells were similar to those observed in insulin-treated pregnant women with T2D, with the exception of B cells which increased in GDM compared to pregnant women with T2D and control pregnant women. In fact, our results were supported by those of Angelo et al. [20] and Mahmoud et al. [48] who have also observed high percentages of CD4+CD25+ and CD4+Th17 cells in insulin-treated women with GDM, suggesting that insulin treatment did not influence the proportion of these cells in pregnant women with in type 2 diabetes as well as in women with GDM [49].

In addition, the decrease of cellular immunity observed in nonpregnant women with T2D treated with insulin was concomitant with the increase of the humoral immunity, consistent with a high number of B cells in these patients [33]. This is not the case in pregnant women with T2D treated with insulin as in the present study. However, the proportion of B cells increased in women with GDM, suggesting an increased humoral immunity during GDM, although the cellular immunity increased. This finding was in accordance with previous results which reported that GDM was associated with an increased maternal humoral immune response against paternal HLA antigens expressed by the fetus [50]. Besides, other researchers have very recently confirmed that the percentage of B lymphocytes was positively associated with insulin resistance in GDM [51].

Interestingly in the present study, high frequencies of total CD3+ and CD4+ T cells were concomitant with high serum IL-2 and IFN-*γ* concentrations observed in insulin-treated pregnant women with T2D and women with GDM compared to pregnant controls. In fact, being a T cell growth factor, IL-2 could induce the increase of T cells by its autocrine action [52]. High production of IFN-*γ* and low level of IL-4 confirmed the proinflammatory Th1 phenotype in insulin-treated T2D pregnant women as well as in women with GDM in this study. Our results are in agreement with those of Seck et al. [4] who reported the proinflammatory status in pregnant diabetic women, which could be responsible of recurrent miscarriage and spontaneous abortion [3].

The anti-inflammatory effect of insulin has been also revealed in our previous study through the increase of serum IL-10 level in T2D patients without pregnancy treated with insulin [33]. In the present study, the serum IL-10 level was lower in insulin-treated T2D pregnant women and women with GDM compared to pregnant controls. In fact, the present results showed that, despite its anti-inflammatory and immunoregulatory properties [26–29, 31], insulin did not induce an anti-inflammatory effect on T2D pregnancy or GDM. Similar results have been obtained by Seck et al. [4] who have observed, at a transcriptional level, a decrease of IL-10 mRNA expression in obese gestational diabetic mothers without insulin treatment, suggesting that insulin had no effect on this cytokine in diabetic mothers [4]. Such observations could be explained by a strong correlation between low systemic IL-10 levels and hyperinsulinemia and insulin resistance or by impaired suppressive activity of regulatory T cells in insulin-treated T2D pregnant women and in women with GDM [53, 54]. Again, low IL-10 levels, confirming decreased suppressive activity of Treg cells, were consistent with Th1 proinflammatory phenotype and increased cellular immunity in these patients. However, it is important to recognize that other studies have reported a downregulation of Th1 cytokines in GDM [1]. Be that it may, our current findings showed that insulin could not induce the shift of Th1/Th2 balance to an anti-inflammatory Th2 phenotype in T2D pregnant patients, as we had previously observed in nonpregnant T2D patients [4]. However, the proinflammatory phenotype associated with GDM was offset by the increased humoral immunity in women with GDM [50, 51].

The T cell functional test performed by in vitro stimulation assay of T cells showed similar proliferative capacity of T lymphocytes and similar secretion of IL-2 and IFN-*γ* in pregnant women, regardless of GDM and T2D, suggesting that lymphocyte reactivity was not affected neither by GDM nor by insulin-treated T2D. Moreover, the mitogen did not increase IL-10 secretion in the supernatants of T cell culture in women with GDM, although it increased its secretion in insulin-treated T2D pregnant women and pregnant controls, despite the no difference in the proportion of regulatory T cells between the three groups. These data were supported by previous studies which showed that the percentage of total CD4+CD127low+/−CD25+FoxP3+ Treg cells was not affected in women with GDM although immune-suppressive activity of these cells reduced as observed in these patients compared to pregnant controls [53]. This may also explain the low IL-10 secretion by PBMCs in women with GDM. In fact, it has been reported that high IL-10 production capacity could confer protection against insulin resistance and T2D [55].

As well as innate immunity cells are concerned, human blood monocytes can be classified into three subsets: classical monocytes (CD14++CD16-), intermediate monocytes (CD14++CD16+), and nonclassical monocytes (CD14+CD16++) [56]. Since monocytes which express CD16 represent a sensitive marker of inflammation or cellular activation, the role of these cells in various diseases has considerable interest in recent years [57–60]. In the present study, the frequencies of classical monocytes decreased while those of nonclassical monocytes increased without any change in intermediate monocytes in women with GDM and insulin-treated T2D pregnant women, compared to control pregnant women. In fact, contradictory results have been obtained by other researchers who have observed an increased percentage of total and classical monocytes in women with GDM compared to the controls [19]. This discrepancy may be explained by the proinflammatory status of GDM women [4], since nonclassical monocytes are involved in the production of inflammatory cytokines [61]. Similar results have been reported in type 2 diabetic patients without pregnancy [60–62], suggesting that pregnancy did not affect the number of these cells.

In summary, our study showed that, although treated with insulin, T2D pregnant women displayed a proinflammatory status consistent with high proportions of CD3+ and CD4+ T cells, upregulation of Th1 cytokines, and low IL-10 production, suggesting a reduced immune-suppressive activity of regulatory T cells. However, GDM, showing proinflammatory status, was associated with activation and maintenance of humoral immunity consistent with high proportion of CD19+ B cells. Further investigations are needed to better understand the lack of response to insulin in T2D pregnancy and the clinical implications of theses immunological parameters in the monitoring of complications associated with diabetes during pregnancy.

## Figures and Tables

**Table 1:** Anthropometric and biochemical data of subjects.

	Pregnant controls *n* = 25	Women with GDM *n* = 15	Insulin-treated T2D pregnant women *n* = 20
Age of subjects			
Mean	29.19 ± 3.94	30.6 ± 3.04	33 ± 3.38
Minimum-maximum	25-39	26-37	29-38
Gestational age (weeks)	28-32	28-35	28-36
Fasting glucose (g/L)	0.80 ± 0.03	1.23 ± 0.05^*αβ*^	0.89 ± 0.02
HbA1c (%)	5.62 ± 0.27	6.58 ± 0.53	5.90 ± 0.1
Duration of disease (years)	—	—	3.4 ± 2.1

HbA1c: glycosylated hemoglobin. Values are means ± SEM. *n* = 15 women with GDM, *n* = 20 insulin-treated T2D pregnant women, and *n* = 25 pregnant controls. ^*α*^*p* < 0.001 indicates significant difference between pregnant controls and women with GDM. ^*β*^*p* < 0.001 indicates significant difference between women with GDM and insulin-treated T2D pregnant women.

**Table 2:** Ratios of serum Th1 and Th2 cytokine concentrations of subjects.

	IL2/IL4	IFN-*γ*/IL4
Pregnant controls	1.38	3.21
Women with GDM	6.13^∗^	6.92^∗^
Insulin-treated T2D pregnant women	4.08^∗^	7.15^∗^

## Data Availability

The data used to support the findings of this study are available from the corresponding author upon request.
